# Compaction Characteristics and Expansion Mechanism of Circulating Fluidized Bed Bottom Slag for Subgrade Filler

**DOI:** 10.3390/ma19214592

**Published:** 2026-10-29

**Authors:** Yong Wang, Peng Gao, Zhida Li, Jianguo Zhang, Zhibing Gao, Mingkai Zhou, Xiaoling Wang

**Affiliations:** 1 Shanxi Luqiao Group Lvliang National Road Project Construction Management Co., Ltd., Lvliang 033000, China; 2 Shanxi Coal Measures Solid Waste Building Materials Resource Utilization Technology Innovation Center, Changzhi 046000, China; 3 School of Materials Science and Engineering, Wuhan University of Technology, Wuhan 430070, China

**Keywords:** circulating fluidized bed bottom slag, unconfined compressive strength, compaction characteristics, expansion mechanism, solid waste valorization

## Abstract

Circulating fluidized bed combustion bottom slag exhibits sand-like particle gradation and self-cementing potential, yet severe vertical expansion restricts its broad engineering application. This study investigates the compaction characteristics and expansion mechanisms of CFBBS using Proctor compaction, unconfined compressive strength (UCS), California bearing ratio (CBR), and expansion tests, combined with X-ray diffraction (XRD), scanning electron microscopy (SEM), and particle-size analysis. Results show that CFBBS has a high optimum moisture content of 15.4–19.6% due to its porous microstructure, and soil incorporation improves its compaction performance. Expansion behavior is synergistically governed by f-CaO and SO_3_ content, curing regime, compaction degree, and soil proportion. Seven-day standard curing, 93% compaction degree, and 20% blended soil provide favorable comprehensive performance with satisfactory mechanical properties and mitigated expansion. A 400 m field trial validates this integrated solution, with no expansion-induced cracks observed and reduced construction cost. This work provides theoretical guidance for the safe reuse of CFBBS as subgrade fill.

## 1. Introduction

Annual global output of coal combustion by-products exceeds one billion tons. Wastes from circulating fluidized bed (CFB) combustion constitute a large and steadily growing fraction of these materials. CFB combustion technology has gained widespread attention owing to its broad fuel flexibility and inherent in-furnace desulfurization capacity [1]. Nevertheless, the physicochemical properties of CFB combustion ashes and slags differ markedly from those of conventional pulverized coal fly ash [2,3]. CFB combustion residues mainly comprise CFB fly ash and CFB bottom slag (CFBBS). Unlike conventional fly ash dominated by smooth spherical glassy particles, CFB residues consist of irregular, highly porous grains with high water absorption. They also contain considerable quantities of free calcium oxide (f-CaO) and type II anhydrite (II-CaSO_4_) [4]. While these features facilitate self-cementing and pozzolanic reactions, they simultaneously lead to high water demand and inferior volume stability if the residues are adopted in conventional cement-based or concrete systems [5,6,7].

Currently, most CFB combustion residues are disposed of via landfilling, imposing heavy environmental loads and causing severe resource waste. It is therefore critical to develop large-scale, cost-effective recycling routes. Benefiting from its relatively coarse grains, continuous sand-like gradation, and promising self-cementing performance, CFBBS is a candidate material for earthworks [1,4,8,9,10,11,12]. Before widespread field implementation, however, two key engineering properties require thorough clarification: compaction behavior and expansion mechanisms. Compaction characteristics govern whether target density can be achieved during construction and lay the foundation for subsequent mechanical performance. In contrast, vertical expansion directly affects the long-term stability of geostructures and constitutes the primary barrier restricting practical utilization [13,14].

Compaction behavior represents a fundamental characteristic of fill materials. Due to the loose porous structure and rough surface texture of CFBBS particles, its compaction response differs significantly from conventional granular fills [4,15,16]. The maximum dry density and optimum moisture content of boiler slag are affected by particle size distribution and fineness modulus. Nevertheless, systematic studies concerning the compaction characteristics of CFBBS from diverse origins are still limited. In engineering practice, cohesive soil is often blended with CFBBS to optimize particle-size gradation and compaction performance [4]. Hence, the effect of soil blending ratio on the compaction properties of CFBBS–soil mixtures deserves further exploration.

Suppressing vertical expansion represents a core scientific challenge for the engineering application of CFBBS. The hydration of f-CaO and sulfate phases within CFBBS produces expansive phases such as ettringite (AFt) and gypsum, triggering vertical expansion [17,18]. Mild expansion may compensate for settlement in compacted fills, yet excessive heave induces cracking and structural failure. Existing literature largely concentrates on the expansion characteristics of CFB fly ash [3,6,7,17,18,19,20,21,22,23,24], while systematic investigations targeting CFBBS remain inadequate. The factors dominating CFBBS expansion and the interplay between expansion driving forces and confinement effects, as well as expansion control strategies based on mixture design and construction parameter optimization, have not been systematically clarified.

Against this background, this study aims to fill the aforementioned research gaps by systematically exploring the compaction characteristics and vertical expansion mechanisms of CFBBS. Firstly, the physical and chemical properties of CFBBS are characterized to clarify the dominant factors affecting its compaction performance. Cohesive soil is blended to optimize the particle gradation and compactability of CFBBS, and the modification mechanisms of the soil blending ratio are comprehensively analyzed. Secondly, the independent and coupled effects of curing condition, calcium–sulfur content, compaction degree, and soil blending ratio on the mechanical performance and volumetric stability of compacted CFBBS are quantitatively evaluated. Microstructural characterization is further conducted to reveal the intrinsic expansion driving forces and matrix confinement mechanisms.

The novelty of this work lies in the integrated investigation of compaction behavior and expansion characteristics of CFBBS. The relative contributions of key influencing factors on vertical expansion are quantified, and targeted optimization strategies are proposed by balancing reduced expansion driving forces and enhanced structural confinement. This study offers experimental evidence and technical references for the large-scale engineering application of CFBBS as fill material in subgrade and earthwork construction.

## 2. Materials and Methods

### 2.1. Raw Materials

Five CFBBS samples (denoted CFBBS1–CFBBS5) were collected from five different thermal power plants across Shanxi Province, China. The particle-size gradation and fineness modulus of the slag samples are summarized in Table S1. The slags exhibit sand-like continuous gradation; CFBBS4 is classified as medium sand, whereas the remaining samples fall into the fine-sand category. Chemical compositions obtained via X-ray fluorescence (XRF) and chemical titration are dominated by SiO_2_ (39.2–46.6%) and Al_2_O_3_ (27.6–29.3%), with considerable contents of SO_3_ (4.35–7.19%) and free-CaO (f-CaO) (2.83–5.46%), as listed in Table S2. X-ray diffraction (XRD) analysis identifies amorphous phases, quartz, II-CaSO_4_, and lime (CaO) (Figure S1a–e). The low-liquid-limit clay (CL) used for soil modification possesses a plasticity index of 10.4 and a CBR of 10.7% at 93% compaction, and it was sourced from the same site as the field trial section. CFBBS5 was chosen as the main raw material in this work. Tests concerning curing regimes, compaction degrees, soil blending ratios, particle breakage, and SEM observations, as well as the field trial, were all conducted on CFBBS5. CFBBS1–CFBBS5 were merely used in Section 3.3 for comparative analysis of CFBBS with varying calcium–sulfur contents from different power plants.

### 2.2. Test Methods

#### 2.2.1. Compaction Test

Compaction tests were carried out following the heavy compaction procedure (modified Proctor) specified in Chinese specification JTG 3430-2020 [25] to determine the optimum moisture content and maximum dry density of CFBBS. Each sample was compacted in five layers inside a standard Proctor mold (internal diameter: 152 mm, volume: 2177 cm^3^). A 4.5 kg rammer falling from a height of 457 mm was adopted, with 98 blows applied to each layer.

#### 2.2.2. Unconfined Compressive Strength (UCS) Test

UCS tests were performed on cylindrical specimens (Φ100 mm × 100 mm) prepared in accordance with JTG E51-2009 [26] (Figure S2). Excluding tests designed to investigate curing regimes, all specimens were sealed with plastic film immediately after compaction and subjected to standard curing at 20 ± 2 °C with relative humidity ≥ 95% until the target testing age.

Specimens for natural curing were stored in a constant-humidity drying chamber maintained at 20 ± 2 °C and 40% relative humidity. To simulate saturated service conditions, selected specimens were immersed in water for 24 h right before testing, and the UCS was then measured. All UCS tests were implemented on a universal testing machine (WDW-100, Changchun Kexin Test Instrument Co., Ltd., Changchun, China) under a constant loading rate of 1 mm/min, complying with the requirements of JTG E51-2009 [26].

#### 2.2.3. California Bearing Ratio (CBR) and Expansion Tests

CBR tests were adopted to acquire three indices: CBR value, water absorption, and expansion ratio (Figure S3). CBR specimens were fabricated by static compaction according to JTG 3430-2020 [25].

Unless stated otherwise for curing regime analysis, specimens for CBR measurement underwent 7 days of standard curing, followed by 4 days of water soaking. A calibrated loading frame was used for penetration testing at a rate of 1 mm/min. The CBR value was defined as the ratio between the measured penetration load and the standard load at a penetration depth of 2.54 mm (or 5.08 mm if the latter yielded a larger value), which characterizes the bearing capacity of compacted CFBBS.

Water absorption was calculated based on the mass increment of CBR specimens after soaking, expressed as the mass percentage of absorbed water relative to the dry specimen mass. This index serves as an indirect indicator of matrix compactness.

The same CBR specimens were used to monitor expansion behavior. A dial gauge with 0.01 mm precision was mounted on the specimen surface to record height variations throughout curing and soaking (Figures S4a and S5b). The expansion ratio was calculated as the percentage change in specimen height relative to the initial dimension. CFBBS specimens were continuously standard-cured until volumetric equilibrium. The expansion ratio reflects the volumetric stability of compacted materials.

It should be noted that CFBBS behaves as a self-cementing weakly cemented material, which differs from ordinary unbound granular materials. The high CBR values obtained under laboratory curing conditions originate from internal hydration-induced strength development and cannot be directly regarded as the immediate bearing capacity after on-site construction.

#### 2.2.4. Quantification of Particle Breakage

The extent of particle breakage within CFBBS–soil mixtures was evaluated by comparing particle size distributions obtained before and after static compaction. Particle size analysis was conducted via the wet-sieving method in accordance with JTG 3430-2020 [25].

Residual mass retained on each sieve (4.75 mm, 2.36 mm, 1.18 mm, 0.6 mm, 0.3 mm, 0.15 mm, and 0.075 mm) was recorded for both uncompacted raw materials and compacted specimens to reflect grading evolution.

In this study, the change in gradation was used to characterize particle breakage. The retained mass on each sieve before and after compaction reflects the effect of static compaction on each size fraction. The “total gradation variation ratio” is defined as the sum of absolute differences in the percentage retained on each sieve before and after compaction, representing the overall disturbance of the gradation caused by static compaction. The equation is:

(1) $$R_{gc}=\sum_{i=1}^{n}\left|p_{i,0}-p_{i,1}\right|$$

where $R_{gc}$ is the total gradation variation ratio (%), $p_{i,0}$ and $p_{i,1}$ are the percentages retained on sieve *i* before and after compaction, respectively, and n is the number of sieve fractions.

#### 2.2.5. Microstructural Analysis

Specimens for microstructural characterization were cured to target ages under designated curing schemes. Material was sampled from the central region of each specimen to guarantee representativeness. The sampled fragments were immediately immersed in anhydrous ethanol for 3 days to terminate ongoing hydration. Afterwards, samples were dried in a vacuum oven at 50 °C for 48 h and preserved in a desiccator prior to testing.

An X-ray diffractometer (Bruker AXS, D8 Advance, Ettlingen, Germany) with Cu Kα radiation (λ = 1.5406 Å) was used to identify crystalline phases and hydration products of compacted CFBBS. The scanning 2θ range was 5–70°, with a scanning speed of 5°/min; the instrument operated at 40 kV and 40 mA. Phase matching was carried out using the ICDD PDF database. Chemical compositions obtained via X-ray fluorescence (XRF) (Zetium, PANalytical, Almelo, The Netherlands).

A scanning electron microscope (JEOL, JSM-IT300, Tokyo, Japan) was utilized to observe the microstructure and morphological features of hydration products. Dried specimens were gold-sputtered before observation to improve surface electrical conductivity. SEM imaging was performed at an accelerating voltage of 15 kV with magnifications ranging from 1000× to 10,000×, enabling simultaneous observation of overall matrix morphology and fine microstructural characteristics of hydration products. Multiple visual fields were observed for each specimen; the presented SEM micrographs are representative of the general microstructural features rather than single-local-field accidental features.

For each test group, 3 parallel replicate specimens were prepared for UCS, CBR, and expansion tests; compaction tests were duplicated twice. All plotted data represent arithmetic mean values of valid replicates. Specimens with obvious manufacturing defects or abnormal failure modes were discarded as outliers, and additional specimens were re-prepared to guarantee valid sample size.

ChatGPT-Image 2 was employed to generate the initial draft of Figure 7. The authors further modified, adjusted, and validated all graphic details of the generated output.

## 3. Results and Discussion

### 3.1. Compaction Characteristics of CFBBS

Compaction characteristics represent the fundamental engineering properties of fill materials, which govern the field compaction feasibility and subsequent mechanical performance of subgrade fills. Due to the unique loose porous structure and rough surface morphology of CFBBS particles, its compaction behavior differs significantly from that of conventional granular fills. This section systematically investigates the effects of slag inherent properties and soil blending ratio on the compaction characteristics of CFBBS, aiming to provide a reliable basis for specimen preparation in subsequent performance tests.

**Figure 1:**
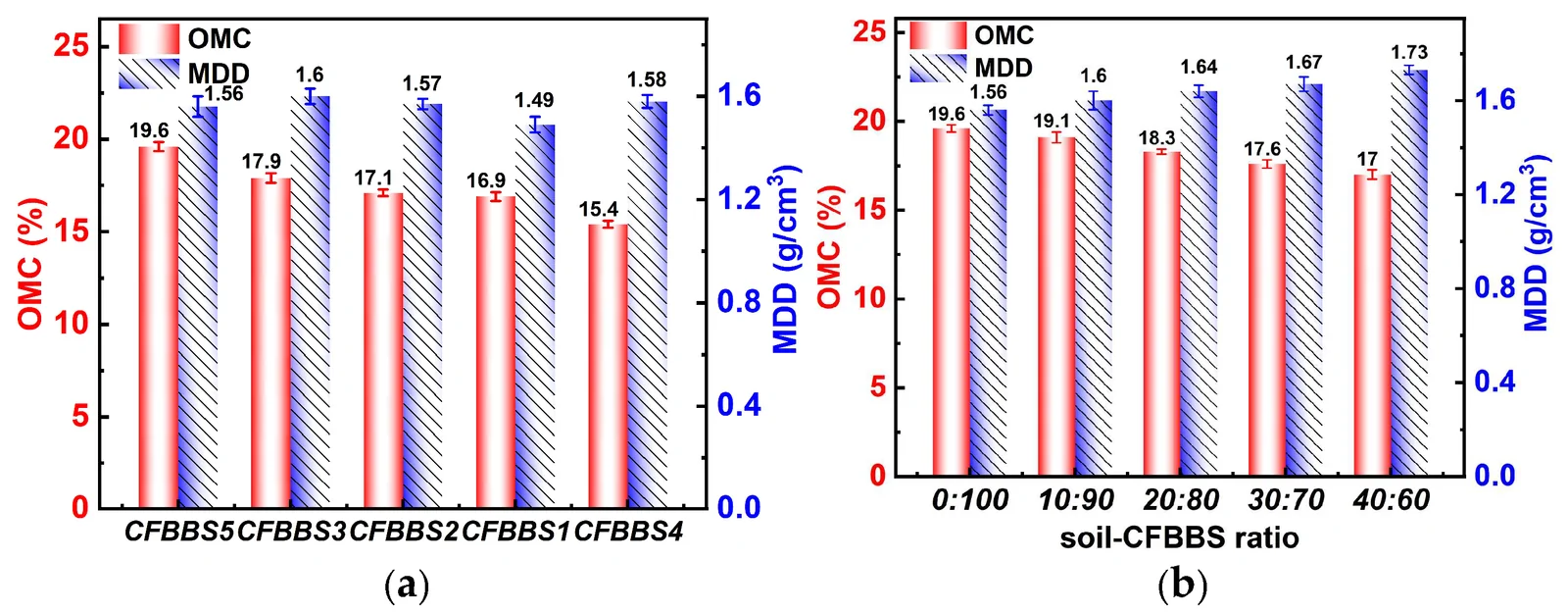
(**a**) Compaction test results of CFBBS with different quality; (**b**) compaction test results of different soil–CFBBS ratio.

Figure 1a illustrates the optimum moisture content (OMC) and maximum dry density (MDD) of five CFBBS samples. The MDD values of different slag samples exhibit minor variations, whereas their OMC differs remarkably, ranging from 15.4% to 19.6%. These values are distinctly higher than the typical OMC range of conventional granular fills (5–10%). A noticeable negative correlation is observed between OMC and fineness modulus (Table S1). Specifically, CFBBS5 presents the lowest fineness modulus (1.58) and the highest OMC (19.6%). This phenomenon is primarily attributed to the large specific surface area of fine particles, which requires more water to form adsorbed water films. Furthermore, the internal porous structure of CFBBS particles allows water to penetrate intraparticle pores during compaction, further increasing the water demand for optimal compaction. In contrast, no obvious correlation is observed between MDD and fineness modulus. Although fine particles can fill interparticle voids and improve matrix compactness, their low particle density restricts the growth of dry density. These two competing effects counteract each other, resulting in a stable MDD across different samples.

Soil blending ratio is the dry-mass percentage of cohesive soil within total dry mass of CFBBS–soil mixture. Figure 1b presents the variations in OMC and MDD of CFBBS–soil mixtures with different soil blending ratios. As the soil blending ratio increases from 0% to 40%, the OMC decreases gradually from 19.6% to 17.0%, while the MDD increases from 1.56 g/cm^3^ to 1.73 g/cm^3^. This evolutionary trend can be explained by two synergistic mechanisms. First, soil exhibits much lower water absorption capacity than porous CFBBS particles. Increasing soil proportion reduces the content of high-water-absorptive slag in the mixture, thereby lowering the overall water demand for optimum compaction. Second, fine soil particles optimize the particle gradation of CFBBS mixtures. Fine soil fractions fill both the intraparticle pores of slag particles and interparticle voids of the matrix, effectively reducing pore volume and ultimately increasing the MDD of the blended mixture.

### 3.2. Effects of Curing Regimes on the Properties of Compacted CFBBS

**Figure 2:**
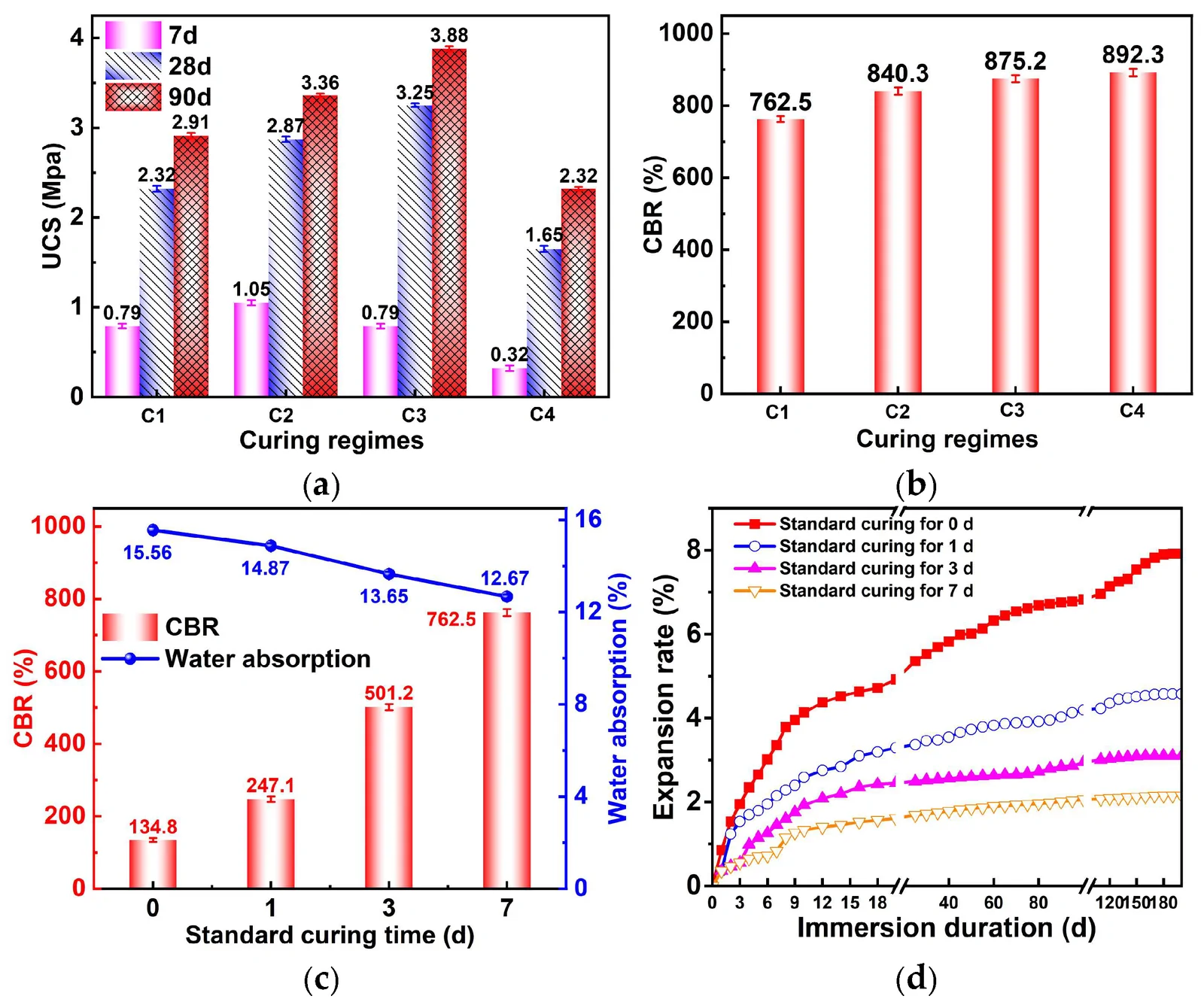
(**a**) Unconfined compressive strength (UCS) under different curing regimes (C1: standard curing (20 ± 2 °C, RH ≥ 95%); C2: natural curing (20 ± 2 °C, RH 40%); C3: standard curing for 7 d followed by natural curing; C4: standard curing, immersed in water for 1 d before the designated age); (**b**) CBR values under different curing regimes (C1: standard curing for 7 d followed by immersion in water for 4 d; C2: standard curing for 11 d; C3: natural curing for 11 d; C4: standard curing for 7 d followed by natural curing for 4 d); (**c**) effects of standard curing duration on CBR values and water absorption; (**d**) effect of immersion duration on expansion ratio.

Curing conditions govern the temporal moisture availability during specimen maturation, thereby regulating the competitive progression of hydration and matrix densification. Accordingly, the curing regime serves as a critical process parameter for controlling the vertical expansion behavior of CFBBS. Figure 2 presents the influences of different curing strategies and standard curing durations on the mechanical performance of compacted CFBBS specimens.

As illustrated in Figure 2a, specimens treated with the C3 curing regime yield the highest UCS at all curing ages. The 28-day UCS reaches 3.25 MPa, which is 40.1% and 31.6% higher than those of the C1 (2.32 MPa) and C2 (2.87 MPa) regimes, respectively. The initial seven-day standard curing supports adequate early-age hydration, which allows ongoing strength gain even under subsequent natural-curing conditions. In comparison, early-stage moisture loss under natural curing restricts hydration activity and slows the strength-gain rate. The C4 curing regime induces the most significant strength degradation, with the 7-day UCS declining by 50.5%. Such strength deterioration cannot be explained merely by moisture softening. Early water immersion generates considerable expansion stress before the matrix develops sufficient structural rigidity, triggering microcrack initiation and irreversible structural damage. This result demonstrates that the timing of water exposure exerts a more dominant effect on structural stability than the total water supply during curing.

Consistent with the strength variation trend, Figure 2b indicates that specimens cured under the C4 regime yield a CBR value of 892.3%, which is 17.0% higher than that of the C1 regime. As shown in Figure 2c, extending the standard curing duration from 0 to 7 days increases the CBR from 134.8% to 762.5%, equivalent to a nearly 5.7-fold increment, while the water absorption rate decreases from 15.56% to 12.67%. The coupled variations in CBR and water absorption are controlled by the same hydration mechanism that dominates strength evolution. Generated hydration products fill internal pores and improve matrix compactness, which effectively enhances the penetration resistance of compacted CFBBS and restricts water infiltration.

Figure 2d presents the effect of standard curing duration on immersion-induced vertical expansion. Specimens subjected to immediate water immersion after molding achieve a maximum expansion ratio of 7.92%, with the expansion process stabilizing at approximately 190 days. In comparison, pre-treatment with 7 days of standard curing prior to immersion reduces the expansion ratio to 2.15% (a 72.8% reduction) and shortens the stabilization period to around 160 days. Such significant expansion mitigation is mainly attributed to the rigid hydration product skeleton formed during pre-curing, which provides effective physical confinement against subsequent expansion stress. Overall, an inverse correlation exists between mechanical strength and vertical expansion. Higher early-age strength offers stronger structural confinement, thereby suppressing the final expansion magnitude. Accordingly, the fundamental function of reasonable curing regime design is to ensure sufficient strength development prior to the occurrence of substantial vertical expansion.

### 3.3. Effects of Calcium and Sulfur Contents in CFBBS on the Properties of Compacted Specimens

**Figure 3:**
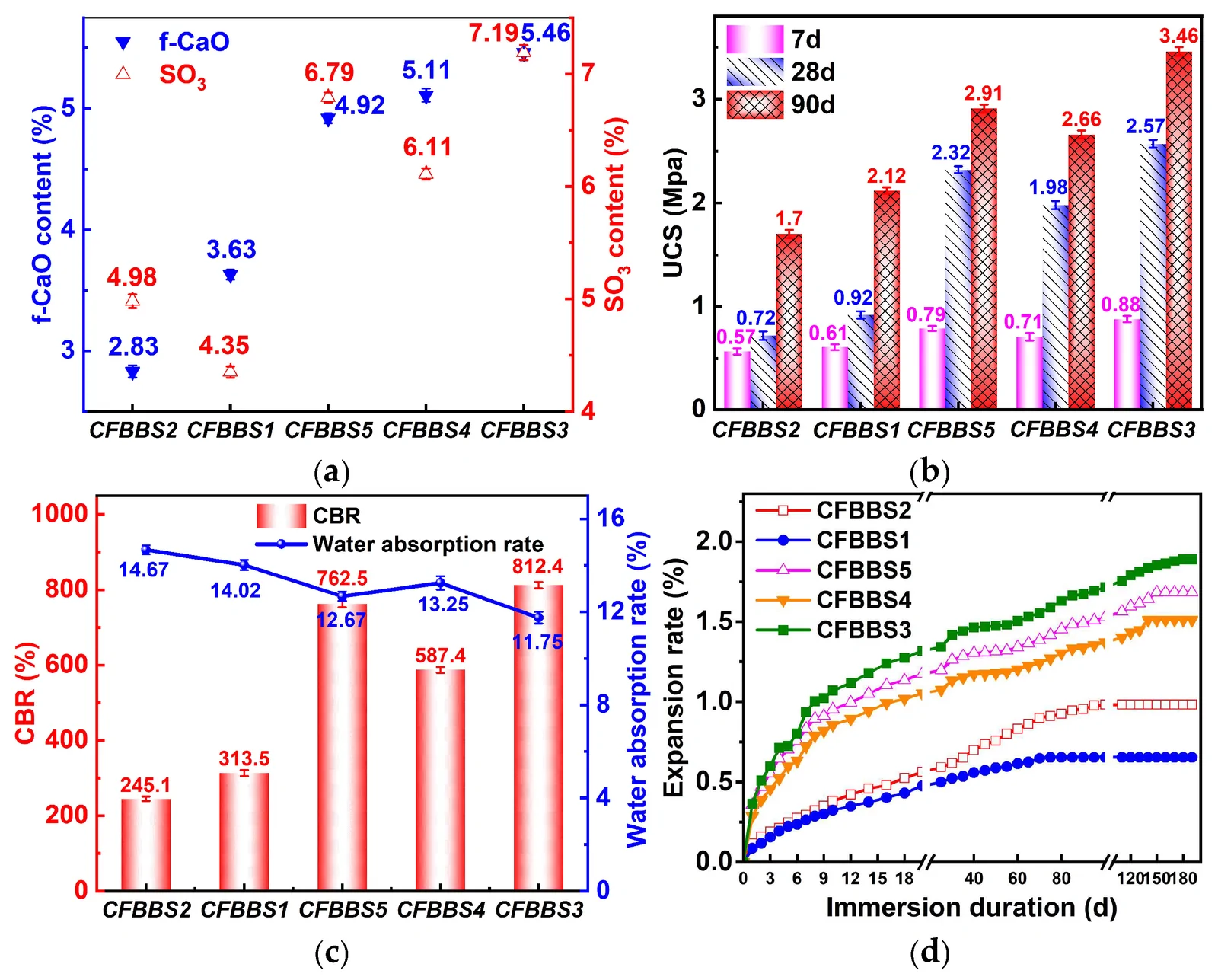
(**a**) f-CaO and SO_3_ contents of CFBBS samples; (**b**) effects of CFBBS quality on UCS; (**c**) effects of CFBBS quality on CBR values and water absorption; (**d**) effects of CFBBS quality on expansion ratio.

CFBBS obtained from different power-plant manufacturers shows considerable variation in f-CaO and SO_3_ contents, as illustrated in Figure 3a. These reactive constituents can modify the types and yields of hydration products and are regarded as potential drivers of vertical expansion. Therefore, exploring correlations between calcium–sulfur chemistry and material performance helps interpret the expansion-related behavior of CFBBS. In this study, five CFBBS samples sourced from different industrial producers were tested. It should be noted that these samples differ not only in f-CaO and SO_3_ but also in particle-size distribution and combustion background, hence the observed trends represent empirical correlations among real-world residues rather than causal relationships from controlled single-variable laboratory tests.

Figure 3b shows UCS results across all curing ages. Slags with higher f-CaO and SO_3_ content tend to deliver greater UCS. Among the tested samples, CFBBS3, which possesses relatively high f-CaO and SO_3_ contents, yields 54.39%, 256.94%, and 103.53% higher UCS at 7 days, 28 days, and 90 days, respectively, compared with CFBBS2. Relative to CFBBS1, CFBBS3 shows 44.62%, 179.35%, and 63.21% strength increments at the corresponding curing ages. The most pronounced strength gain appears at 28 days, indicating that f-CaO- and SO_3_-associated reactions correlate strongly with intermediate-age strength development of CFBBS.

This behavior can be attributed to three mechanisms. First, higher f-CaO increases the alkalinity of the system, accelerates the depolymerization of [SiO_4_] and [AlO_6_] structural units in the glassy phase, and thereby activates pozzolanic reactivity. Second, SO_3_ reacts with C–A–H to form AFt. The acicular and rod-shaped AFt crystals intergrow with C–S–H gel and provide microstructural skeletal reinforcement. Third, unreacted anhydrite hydrates to form gypsum crystals, which also contribute to cementation. The synergistic contributions of the above hydration and pozzolanic reactions collectively promote the continuous strength improvement of CFBBS with elevated f-CaO and SO_3_ contents [4,27].

As shown in Figure 3c, the CBR value correlates positively, and water absorption rate correlates negatively, with f-CaO and SO_3_ contents among the five CFBBS sources. Relative to CFBBS2, CFBBS3 shows a 231.5% higher CBR and 19.9% lower water absorption. Compared with CFBBS1, CFBBS3 delivers a 159.1% increase in CBR and a 16.19% reduction in water absorption. CFBBS3 reaches a CBR as high as 812.4% with a water absorption rate of only 11.8%. This coupled performance trend can be rationalized: greater f-CaO and SO_3_ contents favor the generation of hydration products, which fill pores and improve matrix compactness. The densified microstructure improves penetration resistance and meanwhile blocks water infiltration pathways.

Meanwhile, the findings reveals a correlation between elevated calcium and sulfur levels and poorer vertical deformation stability (Figure 3d). Samples containing higher f-CaO and SO_3_ tend to attain larger vertical expansion ratios and require longer times to reach deformation stabilization. Specifically, CFBBS3 achieved volumetric stability only after 180 days, with a final vertical expansion ratio of 1.89%. Compared with CFBBS1, CFBBS3 exhibited a 100-day extension in stabilization time and an 89% higher final vertical expansion ratio. Although slag with high calcium and sulfur contents developed higher early-age strength, the corresponding increase in expansion restraint was insufficient to offset the greater expansion driving force. Consequently, the final vertical expansion ratio remained higher for CFBBS with higher calcium and sulfur contents.

### 3.4. Effects of Degree of Compaction on the Properties of Compacted CFBBS

**Figure 4:**
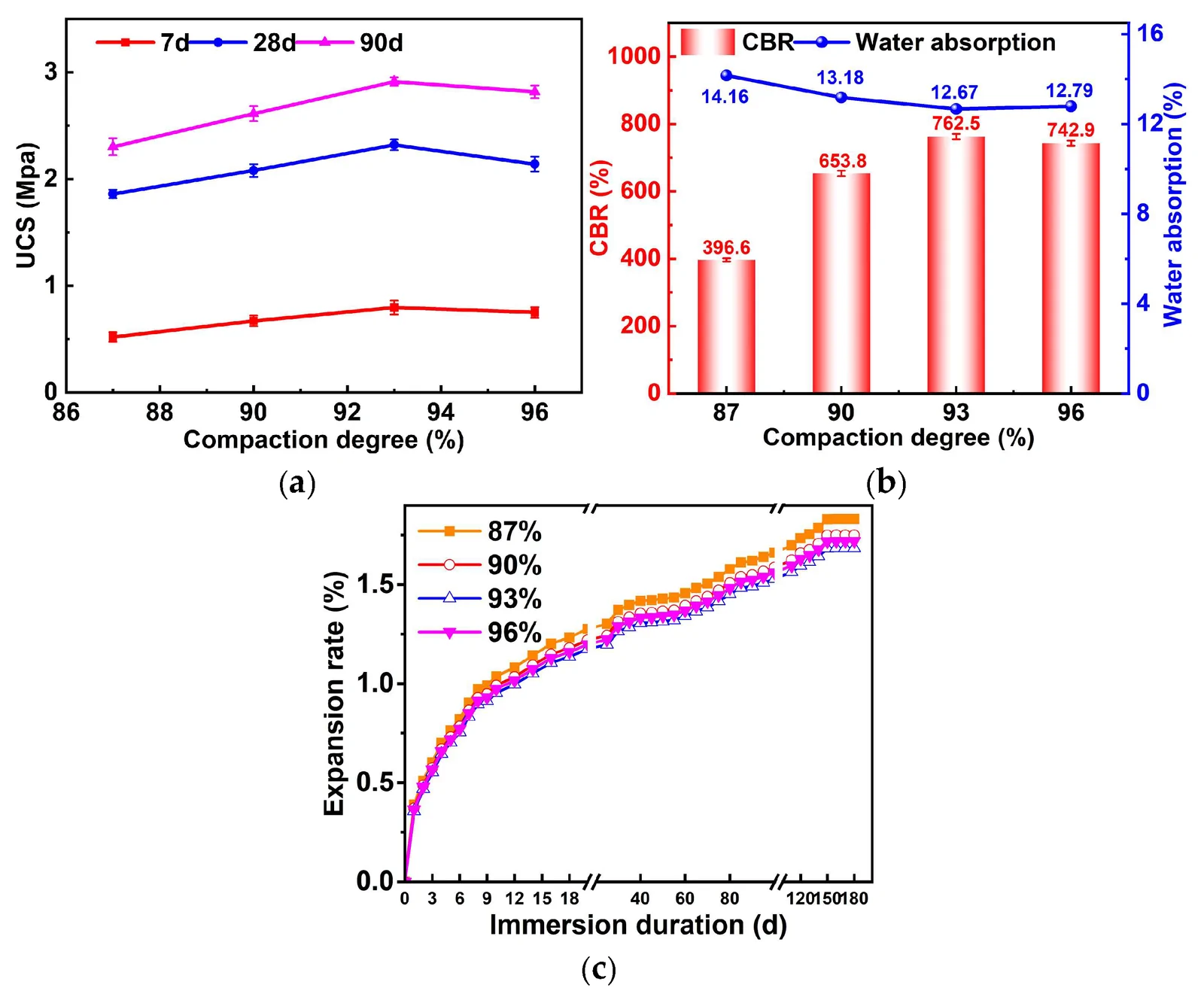
(**a**) Effects of compaction degree on unconfined compressive strength; (**b**) effects of compaction degree on CBR values and water absorption; (**c**) effects of compaction degree on expansion ratio.

Figure 4a reveals that the UCS at all curing ages first rises and then falls as the degree of compaction increases from 87% to 96%, with peak strength achieved at a compaction degree of 93%. For instance, the 90-day UCS of specimens compacted to 93% is 26.5% greater than that of samples compacted at 87%. Nevertheless, further raising the compaction degree to 96% leads to a 7.6% reduction in UCS. This trend correlates with the high particle crushing value of CFBBS. When the compaction degree increases from 87% to 93%, particle rearrangement and tighter packing dominate the compaction process. The growing number of interparticle contact points enables hydration products to bind adjacent particles into an integral matrix, contributing to strength enhancement. Once compaction exceeds 93%, excessive compactive effort aggravates particle breakage, destroys the initial particle skeleton, and elevates fines content, which contributes to reduced structural strength.

Figure 4b indicates that the variations in CBR and water absorption against compaction degree exhibit consistent trends with the UCS results. At a compaction degree of 93%, the CBR reaches its peak value of 762.5%, whereas water absorption declines to the minimum of 12.67%. Specimens compacted at 87% yield a CBR of merely 396.6% and a relatively high water absorption of 14.16%. When the compaction degree rises to 96%, the CBR drops to 742.9%, accompanied by an increase in water absorption to 12.79%. Moderate compaction enhances matrix compactness and facilitates sufficient development of cementitious bonding derived from hydration products; collectively, these effects improve the bearing capacity. In comparison, particle breakage induced by over-compaction not only degrades structural strength but also increases fine content and generates extra micropores, which consequently elevates water absorption.

Figure 4c shows that the vertical expansion ratio gradually rises with curing time for all compaction degrees and tends to stabilize after approximately 160 days. The expansion ratio is 1.83% at 87% compaction, reaches a numerical minimum of 1.69% at 93% compaction, and subsequently increases to 1.72% at 96% compaction. It should be noted that one-way ANOVA indicates that the difference between 93% and 96% compaction is not statistically significant. Vertical expansion behavior exhibits a negative correlation with strength.

### 3.5. Effects of Soil Blending Ratio on the Properties of Compacted CFBBS

**Figure 5:**
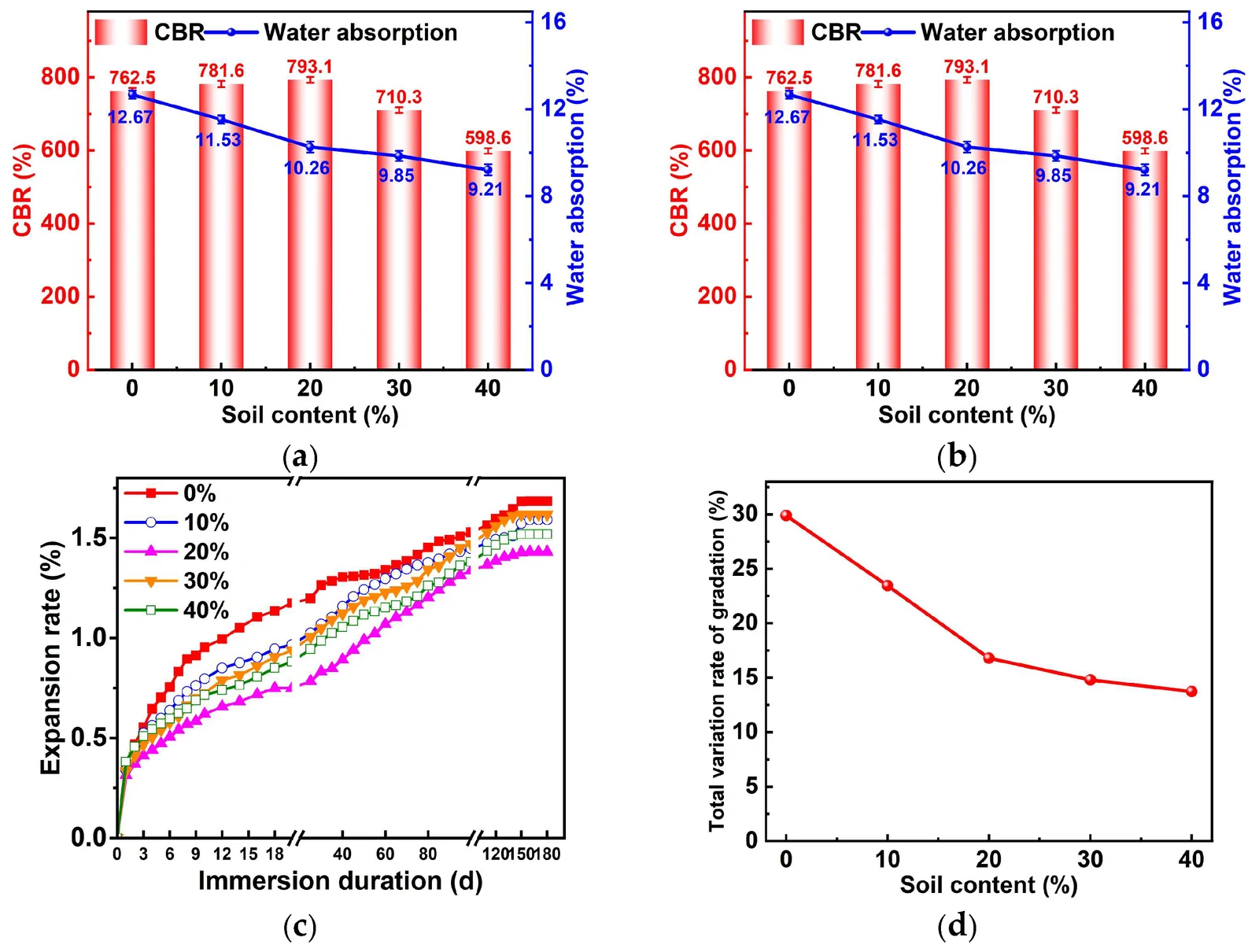
(**a**) Effects of soil content on UCS; (**b**) effects of soil content on CBR values and water absorption; (**c**) effects of soil content on expansion ratio; (**d**) effects of soil content on the total variation rate of gradation before and after compaction.

Incorporation of natural soil is observed to reduce the expansion of CFBBS. However, soil behaves as a relatively inert fraction; higher soil blending ratios correspond to reduced concentrations of reactive components within CFBBS. Figure 5 shows the variations in engineering performance of compacted CFBBS specimens across different soil blending ratios.

As illustrated in Figure 5a, the UCS at all curing ages first rises and then declines when the soil blending ratio increases from 0% to 40%. Peak strength occurs at a soil blending ratio of 20%. Taking the 28-day UCS for comparison, the measured strengths corresponding to soil blending ratios of 0%, 10%, 20%, 30%, and 40% are 2.32, 2.45, 2.59, 1.52, and 1.12 MPa, respectively. The mixture containing 20% soil achieves an 11.6% higher strength than neat CFBBS. By contrast, strength drops drastically once the soil blending ratio exceeds 20%. At a soil blending ratio of 40%, the UCS decreases by 56.8% relative to the 20% blend and is even inferior to that of neat CFBBS. The CBR values shown in Figure 5b followed the same trend as UCS. The maximum CBR, 793.1%, was obtained at a soil content of 20%, whereas it decreased to 598.6% at a soil content of 40%. In contrast, water absorption decreased monotonically with increasing soil content. At a soil content of 20%, the water absorption was 10.26%, representing a 19% reduction relative to that of pure CFBBS. When the soil content increased to 40%, the water absorption further decreased to 9.21%. Figure 5c reveals that the expansion ratio first declines and then rises with increasing soil blending ratio. The minimum expansion ratio of 1.43% is achieved at a soil blending ratio of 20%, corresponding to a 15.4% reduction relative to neat CFBBS (1.69%). This evolutionary trend can be interpreted via two stages. When the soil blending ratio is no higher than 20%, soil particles occupy internal voids and boost matrix compactness; meanwhile, elevated early-age strength increases the matrix’s capacity to confine expansion stress. For soil blending ratios exceeding 20%, the drastic drop in mechanical strength significantly weakens volumetric confinement. Although the generation of expansive products is suppressed, the deterioration of confinement dominates the overall behavior, thereby triggering a rebound in the expansion ratio.

Figure 5d presents the total gradation variation ratio before and after compaction, defined as the summation of absolute differences in mass retained on each sieve between uncompacted and compacted specimens. Figure S5a–e show the mass retained for individual particle size fractions prior to and following compaction. For neat CFBBS, the overall gradation variation ratio reaches 29.88%. As the soil blending ratio increases to 20%, this value declines to 16.79%, equivalent to a 43.8% reduction. Further raising the soil blending ratio to 40% lowers the ratio to 13.74%, yet the reduction rate gradually diminishes. These findings quantitatively verify the buffering effect induced by soil addition. Flexible soil particles dissipate compaction energy, alleviate slag particle breakage, and maintain the integrity of the aggregate skeleton.

**Figure 6:**
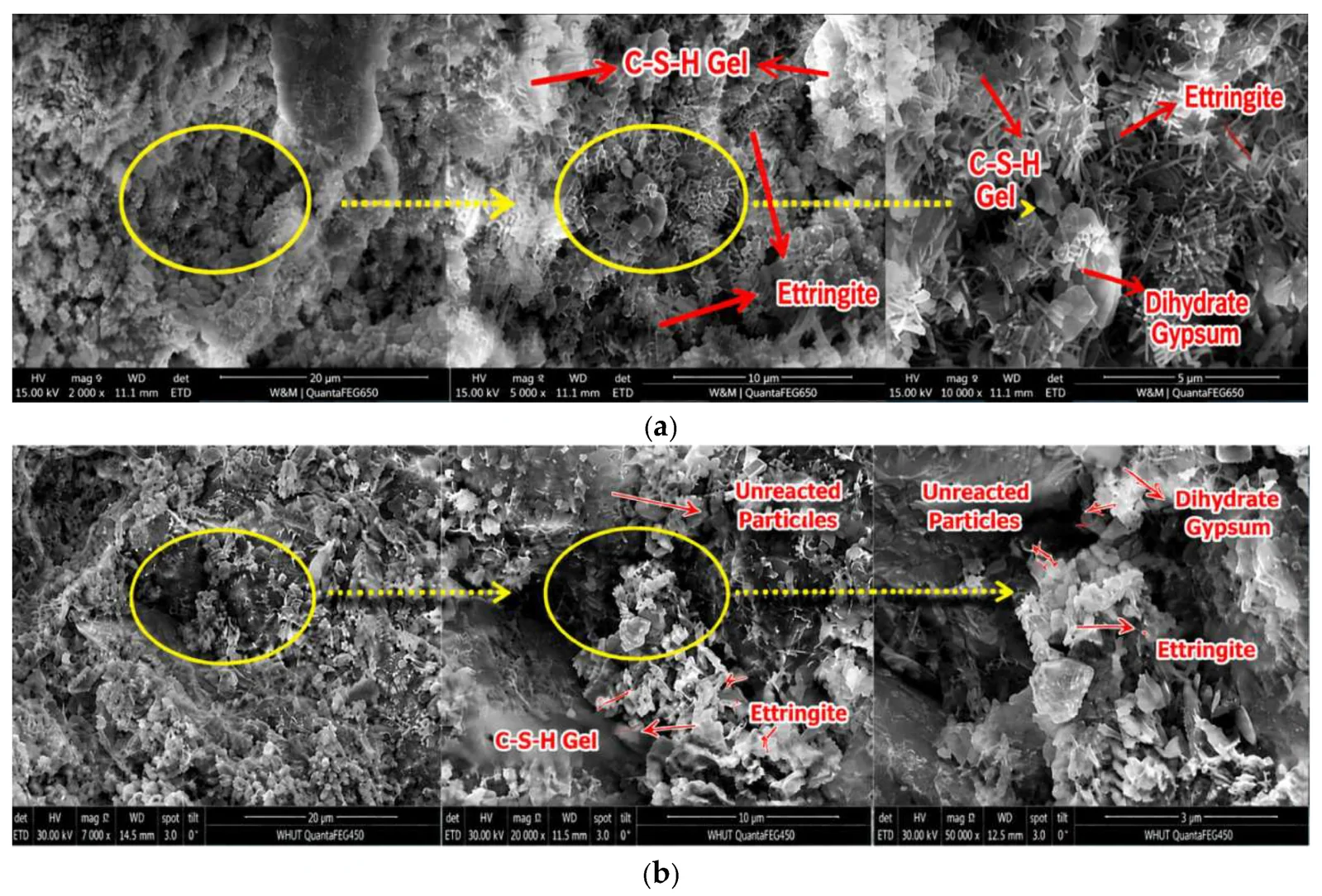
(**a**) SEM image of the CFBBS-based compact without soil addition (0% soil content); (**b**) SEM image of the CFBBS-based compact with 40% soil content. The yellow circles and dashed arrows indicate the regions selected for higher magnification.

The SEM comparison between neat CFBBS (0% soil) and 40% soil-blended mixture is used to qualitatively illustrate microstructural variations induced by soil incorporation. In compacted specimens of neat CFBBS (Figure 6a), abundant flocculent phases and acicular/rod-like crystals are visible on particle surfaces and within interstitial pores. Based on previous literature, these morphologies are characteristic of C–S–H gel and AFt crystals, respectively [15,21].These hydration products are widely distributed on particle surfaces and within interstitial pores, binding nearly all particles into an integral matrix. By comparison, the mixture blended with 40% soil (Figure 6b) exhibits a remarkable reduction in the quantity of hydration products. A large number of unreacted particles remain exposed, and interparticle connections are dominated by point contacts accompanied by insufficient cementitious bonding. Such microstructural features correlate with the pronounced strength reduction observed at high soil blending ratios.

Overall, for soil blending ratios ≤ 20%, the particle buffering effect dominates the material performance, contributing to enhanced mechanical strength and suppressed vertical expansion. In contrast, the dilution effect becomes predominant at soil blending ratios exceeding 20%, which triggers a significant strength degradation and a rebound in expansion deformation.

### 3.6. Schematic Mechanism for Expansion Control and Balance Regulation of CFBBS

**Figure 7:**
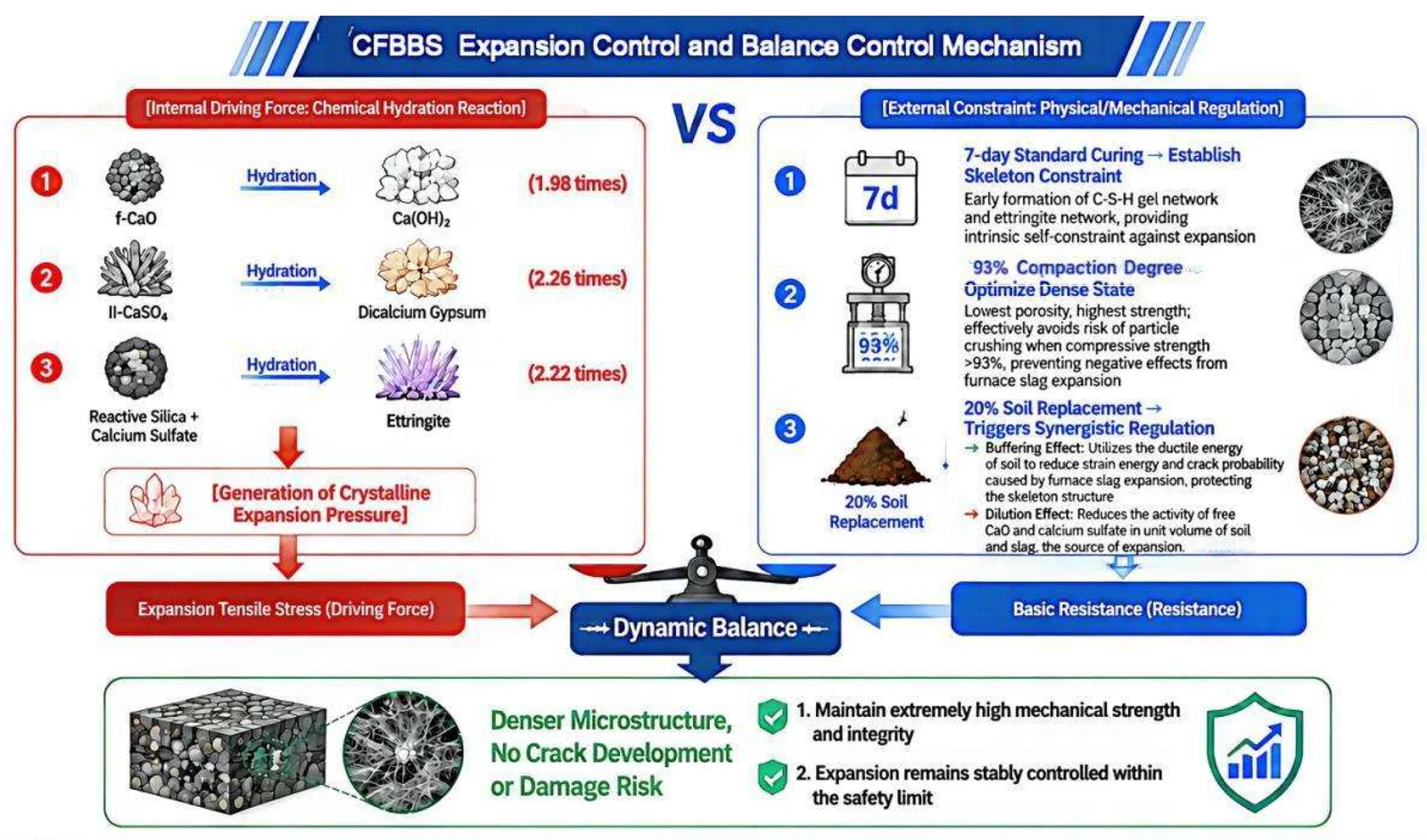
Schematic diagram of expansion control and balance regulation mechanism of CFBBS.

As demonstrated in Figure 7, the vertical expansion of CFBBS originates from volume augmentation accompanying the formation of hydration products. At the early hydration stage, expansion mainly stems from the rapid hydration of f-CaO and II-CaSO_4_, generating Ca(OH)_2_ and gypsum, respectively. The solid volumes of these hydration products reach roughly 1.98 and 2.26 times those of their respective reactants, inducing significant early-age water-triggered expansion of the matrix. During the intermediate and later stages, dissolved Ca^2+^ and SO_4_^2−^ participate in self-pozzolanic reactions with activated metakaolin-like phases on slag surfaces, continuously forming AFt crystals. The precipitation of AFt is accompanied by a volume increase of approximately 2.22 times. Considerable crystallization pressure generated during the growth of AFt and gypsum crystals is hypothesized as the major driving force for the sustained, progressive long-term vertical expansion of hardened CFBBS based on published literature [5,7,28,29,30]. It should be noted that crystallization pressure and quantitative C–S–H content were not directly measured in this study. The inference regarding crystallization pressure and C–S–H-dominated confinement is derived indirectly from mechanical performance together with SEM observations; dedicated quantitative experiments are required for further verification. The ultimate expansion ratio depends on the equilibrium between this expansion driving force and the confinement force. The confinement force is collectively governed by curing condition, compaction degree, and soil blending ratio.

Seven days of standard curing facilitates the preferential development of a rigid C–S–H gel skeleton, which physically confines expansion stresses generated in the subsequent hydration process. A compaction degree of 93% achieves a favorable balance between matrix compactness and aggregate skeleton integrity in terms of mechanical performance. Statistical analysis indicates no significant difference in vertical expansion between 93% and 96% compaction, therefore 93% cannot be regarded as a statistically verified optimum for expansion behavior alone. A soil blending ratio of 20% produces two complementary influences. Firstly, the buffering effect mitigates particle breakage of CFBBS. Secondly, the dilution effect restricts the total quantity of hydration products formed. The coordinated regulation of curing condition, compaction degree, and soil blending ratio enables the expansion driving force to be effectively contained within the confining skeleton, realizing feasible engineering control over the vertical expansion of CFBBS.

### 3.7. Engineering Application and Field Validation

To verify the engineering performance of CFBBS utilized as subgrade fill, a 400 m trial section was constructed at an industrial site in Luliang, Shanxi Province, China. The test section featured a width of 8 m and a filling thickness of 0.74 m, comprising two proportion schemes: 90% CFBBS blended with 10% soil from K0 + 400 to K0 + 600, and 80% CFBBS blended with 20% soil from K0 + 600 to K0 + 800. The target compaction degree was specified as 93%. The fill material was placed in four construction lifts, with an initial loose lift thickness of 250 mm and a loose-compacted thickness coefficient of 1.35%.

Field performance tests included core sampling for UCS (Table S3), Benkelman beam rebound deflection (Table S4), expansion tests on laboratory CBR specimens (Figure S6), and crack monitoring. The test results are summarized in Table 1. All measured indices comply with the design specifications. The 7-day core strength of the mixture consisting of 80% CFBBS and 20% soil is markedly higher than that of the blend with 90% CFBBS and 10% soil, which agrees well with laboratory findings. Strength continuously increases up to 56 days, demonstrating sustained hydration activity at later curing ages. The representative resilient deflection also satisfies the design criteria. Furthermore, the mixture incorporating 20% soil yields a smaller deflection value, reflecting improved overall bearing capacity. The trial section was monitored for cracks for 6 months after construction. Visual inspection and crack width measurements were conducted every two weeks. No expansion-related cracks were detected during the monitoring period. The trial section was open to traffic, mainly heavy construction and transport vehicles. The absence of cracks verifies the reliability of the integrated technical scheme: 20% soil blending ratio, 93% compaction degree, and 7-day moisture-retaining curing.

From an economic perspective, CFBBS is available at zero procurement cost as a waste by-product directly supplied by power plants. Compared with conventional cement-stabilized soil, the adoption of CFBBS subgrade fill reduces the construction cost by 21.6 Chinese Yuan per cubic meter, which is mainly attributed to the elimination of cement consumption required for cement-stabilized soil treatments. The 400 m trial section achieved a total cost saving of 51,000 Chinese Yuan and consumed 3258 tons of waste boiler slag. A detailed cost comparison is presented in Table S5. These outcomes demonstrate the excellent economic efficiency of CFBBS recycling in subgrade engineering. This reuse approach realizes waste diversion and reduces cement demand, with potential environmental benefits; further leaching testing and life-cycle assessment are required for full environmental evaluation.

## 4. Conclusions

This study investigates the compaction characteristics and expansion mechanisms of circulating fluidized bed combustion bottom slag (CFBBS) through laboratory testing and field validation. CFBBS has a high optimum moisture content (15.4–19.6%), attributed to its porous structure; lower fineness modulus corresponds to higher optimum moisture content. Soil addition optimizes compaction properties, yet modification efficiency declines at excessive soil fractions.

Vertical expansion of CFBBS is associated with crystallization pressure during hydration of f-CaO and sulfur-bearing phases, which correlates with intrinsic Ca–S contents and external factors (curing regime, compaction degree, soil proportion). Higher calcium–sulfur contents elevate expansion potential and prolong the stabilization period. The 7-day initial standard curing corresponds to a 72.8% reduction in immersion-induced expansion, which may be linked to confinement provided by the rigid C–S–H skeleton. Unmodified CFBBS exhibits inferior mechanical and deformation performance under long-term water-saturated conditions.

The combination of 93% compaction degree and 20% soil blending ratio achieves favorable comprehensive performance in terms of mechanical properties and vertical deformation stability. The blended soil mitigates particle breakage and reduces the relative content of expansive reactive components in the CFBBS mixture. A 400 m field trial demonstrates that this parameter scheme meets conventional subgrade performance requirements without the occurrence of expansion-induced cracks, verifying the feasibility of CFBBS recycled utilization in subgrade engineering.

## Figures and Tables

**Table 1:** Road performance test results of CFBBS in the field trial section.

Test Index	90% CFBBS + 10% Soil	80% CFBBS + 20% Soil
Compaction degree (%)	93.8	94.7
7-day UCS (MPa)	1.17	1.50
56-day UCS (MPa)	2.70	2.91
Representative value of resilient deflection (0.01 mm)	122.01	118.45
Expansion ratio after stabilization (%)	1.58	1.43

## Data Availability

The data used to support the findings of this study can be made available from the corresponding author upon request.

## Supplementary Materials

The following supporting information can be downloaded at: https://www.mdpi.com/article/10.3390/ma19214592/s1, Figure S1: XRD patterns of samples: (a) CFBBS1; (b) CFBBS2; (c) CFBBS3; (d) CFBBS4; (e) CFBBS5; Figure S2: Bagging maintenance of UCS; Figure S3: CBR test; Figure S4: Monitor expansion behavior of specimens; Figure S5: Influence of soil blending ratio on mass retained of mixtures before and after compaction: (a) 0%; (b) 10%; (c) 20%; (d) 30%; (e) 40%; Figure S6: Expansion rate of CFBBS and soil mixture solidified body; Table S1: particle gradation and fineness modulus for 5 CFBBS samples; Table S2: Chemical composition of CFBBS; Table S3: UCS test results of core samples from the CFBBS trial section; Table S4: Rebound deflection test results of the CFBBS trial section; Table S5: Comparative analysis of economic cost of CFBBS subgrade material.
