# Supplementary material for: Compaction Characteristics and Expansion Mechanism of Circulating Fluidized Bed Bottom Slag for Subgrade Filler
Source: Materials (Basel). 2026 Oct 29;19(21):4592. doi: 10.3390/ma19214592 (PMC13764480; doi:10.3390/ma19214592)
Supplement: Supplementary file 1 [file materials-19-04592-s001.zip › materials-4546820-supplementary.pdf]

Supplementary Materials

Compaction Characteristics and Expansion Mechanism of Circulating Fluidized Bed Bottom Slag for Subgrade Filler

Table S1. Particle gradation and fineness modulus for five CFBBS samples.

	Percentage passing by mass								Fineness modulus
Sieve size (mm)	9.5	4.75	2.36	1.18	0.6	0.3	0.15	0.075	
CFBBS1	98.7	93.4	83.2	74.3	59.7	38.1	20.5	11.0	2.05
CFBBS2	100	98.4	93	84.5	66	39.5	23.7	12.8	1.88
CFBBS3	99.4	89.8	83.4	78.8	70.0	48.8	23.4	11.7	1.61
CFBBS4	97.9	90.4	77.1	66.8	51.9	30.6	14.3	6.8	2.33
CFBBS5	99.8	94.9	88.1	82.5	72.6	52.5	28.7	13.5	1.58

Table S2. Chemical composition of CFBBS.

CFBBS samples	XRF elemental analysis results (%)							Chemical titration results (%)	
	SiO_2	Al_2O_3	Fe_2O_3	CaO	MgO	SO_3	Loss	f-CaO	SO_3
CFBBS1	46.6	29.3	3.6	8.1	0.6	6.4	3.7	3.63	4.35
CFBBS2	43.8	27.9	5.8	7.6	1.1	7.8	6.2	2.83	4.98
CFBBS3	39.2	29.1	4.9	10.5	0.9	9.9	7.1	5.46	7.19
CFBBS4	40.8	28.8	4.7	9.6	1.4	8.4	4.3	5.11	6.11
CFBBS5	41.6	27.6	5.3	9.8	0.8	9.6	4.6	4.92	6.79

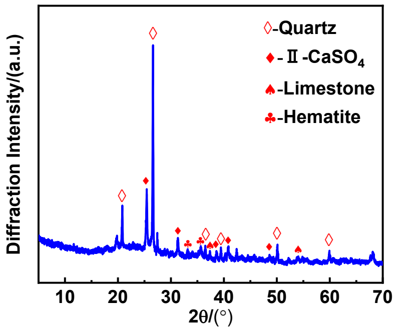

(a)

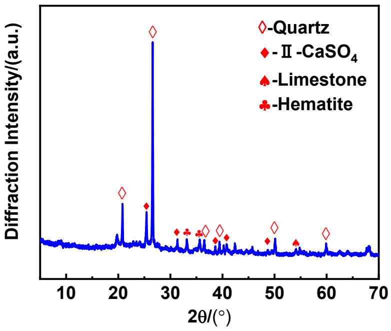

(b)

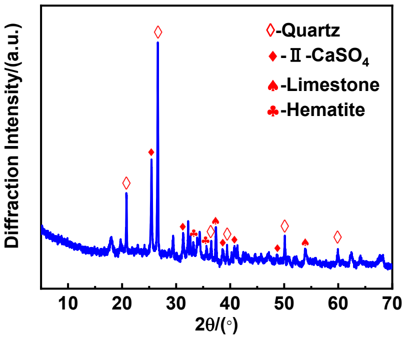

(c)

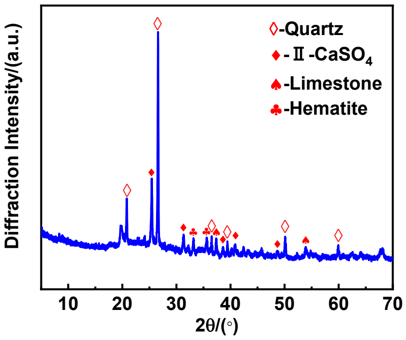

(d)

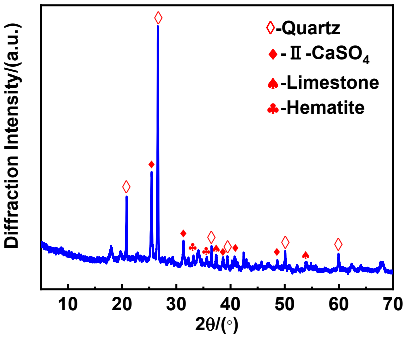

(e)

Figure S1. XRD patterns of samples: CFBBS1(a); CFBBS2(b) ; CFBBS3(c) ; CFBBS4(d) ; CFBBS5(e).

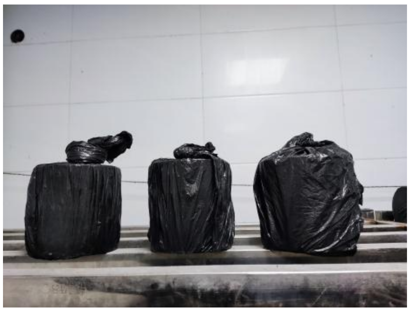

Figure S2. Bagging maintenance of UCS.

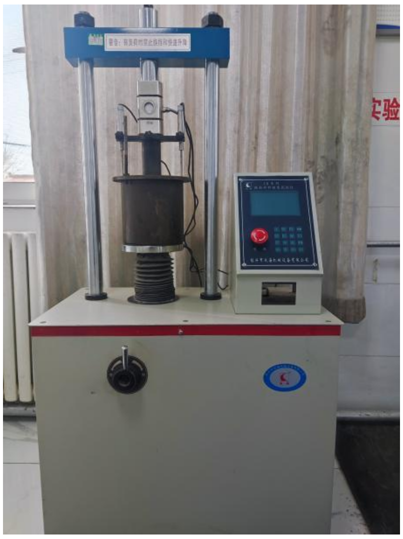

Figure S3. CBR test.

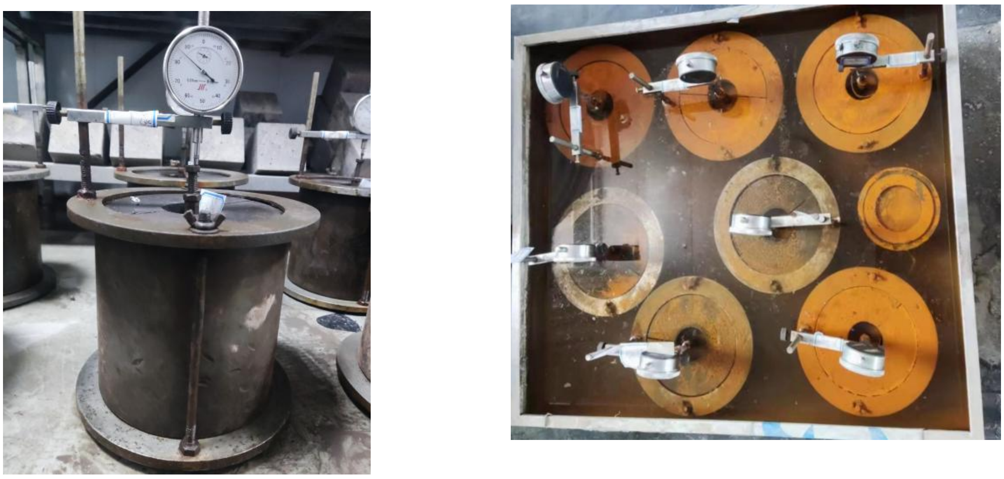

(a) Standard curing (b) Curing immersed in water

Figure S4. Monitor expansion behavior of specimens.

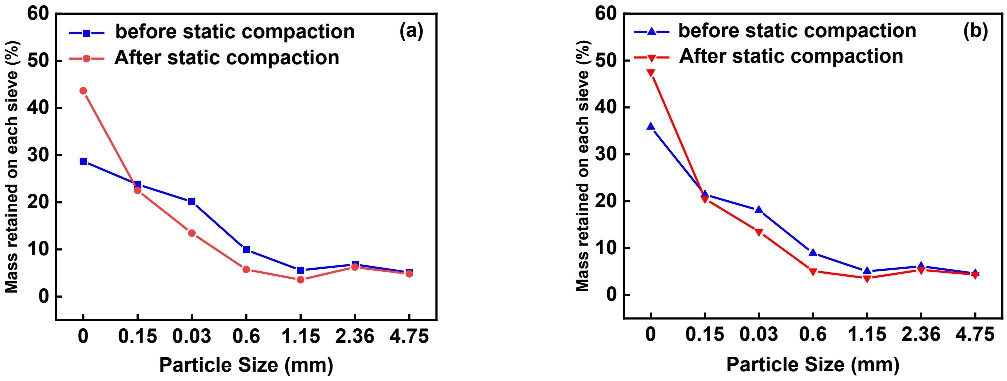

(a) (b)

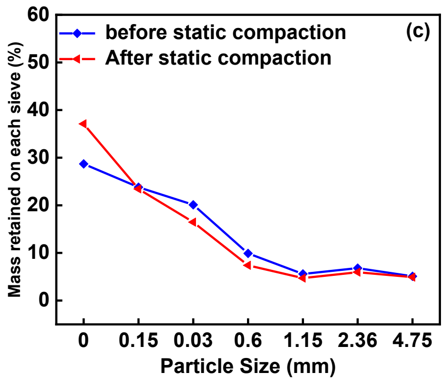

(c)

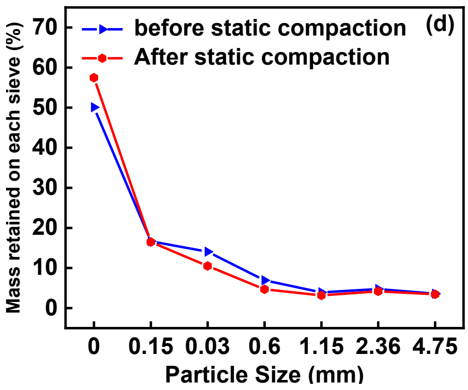

(d)

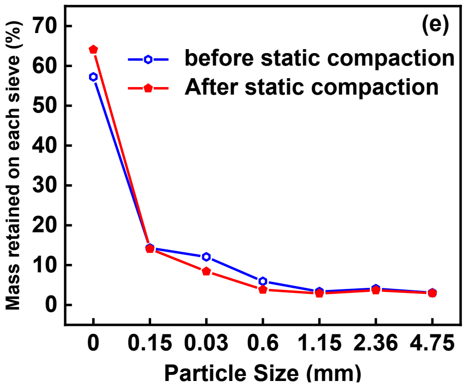

(e)

Figure S5. Influence of soil blending ratio on mass retained of mixtures before and after compaction: (a) 0%; (b) 10%; (c) 20%; (d) 30%; (e) 40%.

Table S3. UCS test results of core samples from the CFBBS trial section.

Test location	UCS			
	7d	Average	56d	Average
K0 + 430	1.26	1.17	2.92	2.70
K0 + 470	1.05		2.61	
K0 + 530	1.24		2.68	
K0 + 570	1.13		2.59	
K0 + 630	1.62	1.50	3.15	2.91
K0 + 670	1.45		2.80	
K0 + 730	1.57		3.09	
K0 + 770	1.36		2.60	

Note: Core sampling and UCS tests. After compaction and curing, core samples were drilled at eight locations: K0 + 430, K0 + 470, K0 + 530, and K0 + 570 for the 90% CFBBS + 10% soil section, and K0 + 630, K0 + 670, K0 + 730, and K0 + 770 for the 80% CFBBS + 20% soil section. At each location, one core sample was drilled, and UCS tests were performed at 7 and 56 days.

Table S4. Rebound deflection test results of the CFBBS trial section.

Test section	Number of points	Average（0.01 mm）	Standard deviation（0.01 mm）	Representative value（0.01 mm）
90% CFBBS + 10% soil	19	105.22	11.90	122.01
80% CFBBS + 20% soil	19	103.75	9.19	118.45

Note: After 7 days of curing, rebound deflection was measured using the Benkelman beam method at intervals of 10 m starting from K0 + 400. A total of 19 test points were selected for each mixture.

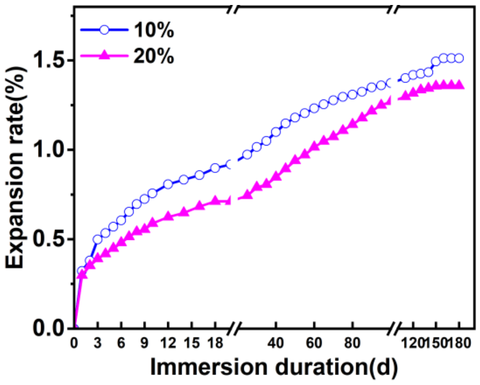

Figure S6. Expansion rate of CFBBS and soil mixture solidified body.

Note: The expansion ratio was determined from laboratory CBR specimens prepared with the same CFBBS–soil mixtures used in the field trial. The specimens were subjected to standard curing, and the expansion ratio was monitored for up to 180 days.

Table S5. Comparative analysis of economic cost of CFBBS subgrade material.

Item	90% CFBBS + 10% soil		80% CFBBS + 20% soil		Cement-stabilized soil (3% cement)	
Compacted bulk density (t/m^3)	1.60		1.64		1.85	
Raw material	CFBBS	soil	CFBBS	soil	cement	soil
Dosage (t/m^3)	1.440	0.160	1.312	0.328	0.054	1.796
Unit price (Yuan/t)	0	0	0	0	400	0
Raw material cost ($Yuan/m^3$)	0	0	0	0	21.6	0
Mixture cost ($Yuan/m^3$)	0		0		21.6	
Cost saving ($Yuan/m^3$)	21.6		21.6		-	

Mixture cost (Yuan)	0	0
Cumulative cost saving (Yuan)		51000

Note: The cost saving of 21.6 Yuan/m^3 is mainly due to the zero procurement cost of CFBBS and the elimination of cement. Transportation, preprocessing, storage, additional water, curing, and quality control costs are not included in this simplified analysis and should be considered in future studies.
